# Lymphocyte subsets in healthy Malawians: Implications for immunologic assessment of HIV infection in Africa

**DOI:** 10.1016/j.jaci.2009.10.010

**Published:** 2010-01

**Authors:** Wilson L. Mandala, Jenny M. MacLennan, Esther N. Gondwe, Steven A. Ward, Malcolm E. Molyneux, Calman A. MacLennan

**Affiliations:** a Malawi-Liverpool-Wellcome Trust Clinical Research Programme, College of Medicine, University of Malawi, Blantyre, Malawi; b Department of Microbiology, College of Medicine, University of Malawi, Blantyre, Malawi; c Liverpool School of Tropical Medicine, University of Liverpool, Liverpool, United Kingdom; d Division of Medical Microbiology, School of Infection and Host Defence, University of Liverpool, Liverpool, United Kingdom; e Department of Zoology, University of Oxford, Oxford, United Kingdom; f Medical Research Council Centre for Immune Regulation and Clinical Immunology Service, School of Immunity and Infection, University of Birmingham, Birmingham, United Kingdom

**Keywords:** Lymphocyte subsets, immunophenotyping, flow cytometry, CD4 lymphocyte count, Africa, HIV, AIDS, ART, Antiretroviral therapy, NK, Natural killer, WHO, World Health Organization

## Abstract

**Background:**

CD4^+^T lymphocyte measurements are the most important indicator of mortality in HIV-infected individuals in resource-limited settings. There is currently a lack of comprehensive immunophenotyping data from African populations to guide the immunologic assessment of HIV infection.

**Objective:**

To quantify variation in absolute and relative lymphocyte subsets with age in healthy Malawians.

**Methods:**

Lymphocyte subsets in peripheral blood of 539 healthy HIV-uninfected Malawians stratified by age were enumerated by flow cytometry.

**Results:**

B and T–lymphocyte and T-lymphocyte subset absolute concentrations peaked in early childhood then decreased to adult levels, whereas lymphocyte subset proportions demonstrated much less variation with age. Adult lymphocyte subsets were similar to those in developed countries. In contrast, high B-lymphocyte and CD8^+^T-lymphocyte levels among children under 2 years, relative to those in developed countries, resulted in low CD4^+^T-lymphocyte percentages that varied little between 0 and 5 years (35% to 39%). The CD4^+^T-lymphocyte percentages in 35% of healthy children under 1 year and 18% of children age 1 to 3 years were below the World Health Organization threshold defining immunodeficiency in HIV-infected children in resource-limited settings. Thirteen percent of healthy children under 18 months old had a CD4:CD8T-lymphocyte ratio <1.0, which is commonly associated with HIV infection. All immunologic parameters except absolute natural killer lymphocyte concentration varied significantly with age, and percentage and overall absolute CD4^+^T-lymphocyte counts were higher in females than males.

**Conclusion:**

Although lymphocyte subsets in Malawian adults are similar to those from developed countries, CD4^+^T-lymphocyte percentages in young children are comparatively low. These findings need to be considered when assessing the severity of HIV-related immunodeficiency in African children under 3 years.

There has been a rapid expansion in the numbers of HIV-infected individuals commencing antiretroviral therapy (ART) in Malawi^1^ and elsewhere in sub-Saharan Africa. Eligibility for ART has been heavily reliant on HIV/AIDS clinical staging criteria,^1,2^ but we and others have shown that this is a poor indicator of immunosuppression.^3,4^ There has been a steady increase in the use of CD4^+^ T-lymphocyte enumeration (CD4 counting) by flow cytometry to guide clinical decision-making in the management of HIV infection in Africa. Although regarded by some as inappropriate in resource-limited settings,^1^ we and others have demonstrated that flow cytometry can be both inexpensive and straightforward to use.^3,5-8^ CD4^+^ T-lymphocyte numbers have been shown to be the most important indicator of mortality in resource-limited settings, particularly among children.^9^

The accurate interpretation of immunophenotyping data, including CD4 counts, is reliant on age-specific lymphocyte subset reference values from healthy individuals in the relevant population. This is especially important for young children, because lymphocyte subsets vary markedly with age during childhood.^10-13^ Well established reference values are available for the developed world^11-14^ but are lacking for resource-limited settings, especially Africa. Previous immunophenotyping reference values from Africa have often had limited subject numbers and broad age groups^15,16^ and been restricted to adult subjects^17-20^ and enumeration of CD4^+^ and CD8^+^ T-lymphocyte subsets.^16,21^

Current World Health Organization (WHO) guidelines for the immunologic assessment of HIV-infected children in resource-limited settings are based on studies from developed countries.^2,22,23^ These studies may not be applicable to the developing world, where genetic and environmental factors, particularly exposure to infectious diseases, are different. In this study we present reference lymphocyte subset ranges derived from 539 healthy individuals from Malawi.

## Methods

### Study design

The study was conducted at the Ndirande Health Centre, Ndirande township, Blantyre, Malawi, between October and December 2005 and September and November 2006. Subjects were healthy volunteers from the township and were made aware of the study by health care assistants at immunization clinics and by local leaders in the community. Each individual was assessed clinically by a research nurse and clinical officer for eligibility to take part in the study. This included a review of each participant's health passport, a medical history, and a clinical examination. In particular, participants were assessed for signs of febrile illness and active infection, malnutrition, and clinical AIDS. Participants were asked whether they were on any medication and whether they had a history of any illnesses. Approximately 50 subjects were recruited into each of 11 groups according to age: 0 to 6, 6 to 12, 12 to 18, and 18 to 24 months, and 2 to 3, 3 to 5, 5 to 10, 10 to 15, 15 to 20, 20 to 60, and over 60 years. After informed consent, baseline demographic data, height, weight, and a 5-mL blood sample were obtained. A follow-up appointment was offered 1 week later to give results. HIV-infected adults and children were referred to the ART clinic.

### Investigations and flow cytometry

Investigations were performed on EDTA-anticoagulated blood on the day of venesection at the Blantyre Malaria Project/Ndirande Health Centre and Malawi-Liverpool-Wellcome Trust Clinical Research Programme. Two HIV rapid tests, Determine (Abbott Laboratories, Tokyo, Japan) and UniGold (Trinity Biotech, Dublin, Ireland), were performed. Positive results in children under 18 months and discordant results were confirmed by PCR as previously described.^24^ A malaria parasite slide was prepared, and full blood count and white cell differential were performed on a HMX hematologic analyzer (Beckman Coulter, Fullerton, Calif).

For immunophenotyping, 25 μL blood was labeled with 4 mAbs in 2 tubes. Tube 1 contained T cell receptor (TCR)-γδ–fluorescein isothiocyanate, CD16/CD56-phycoerythrin, CD3-PerCP, and CD8-allophycocyanin (APC). Tube 2 contained CD4–fluorescein isothiocyanate, CD27-phycoerythrin, CD3-peridin chlorophyll protein (PerCP), and CD19-APC (all Becton Dickson, San Jose, Calif). Red cells were lysed by using FACSlysing solution (Becton Dickinson) and samples washed with PBS. Sample data were acquired by using a FACSCalibur flow cytometer (Becton Dickinson). Daily calibration and internal quality assurance of the instrument was performed by using Calibrite Beads and FACSComp software (both Becton Dickinson). Sample internal quality assurance involved confirming that the sum of T, B, and natural killer (NK) subset percentages was within 100% ± 6%. External quality assurance was undertaken through the United Kingdom National External Quality Assurance Scheme (UK NEQAS) immune monitoring scheme.^25^ Intra-assay and interassay coefficients of variation were 5.2% and 10.3%, respectively, for absolute CD4^+^ T-lymphocyte counts and 2.5% and 7.3% for CD4^+^ T-lymphocyte percentages.

Sample data were analyzed by using CellQuest software (Becton Dickinson). Total lymphocytes were identified by light scatter characteristics and the following lymphocyte subpopulations identified as percentages of the lymphocyte gate: T cells (CD3^+^), CD4^+^ T cells (CD3^+^CD4^+^), CD8^+^ T cells (CD3^+^CD8^+^), γδT cells (CD3^+^TCRγδ^+^), B cells (CD19^+^), memory B cells (CD19^+^CD27^+^), and NK cells (CD3-CD16/56^+^). Lymphocyte subpopulation concentrations were determined from subpopulation percentages and total lymphocyte counts from the hematologic analyzer.

### Exclusions

Subject exclusion criteria were concurrent illness and/or medication, axillary temperature >38 °C, severe malnutrition (weight-for-height < 70% in children or body mass index <16% in adults), unknown date of birth, and known HIV/AIDS. Blood samples were rejected if they were HIV-infected, if malaria parasites were present, or if the sample was clotted or was not processed on the day of venesection.

### Statistical analysis

Medians and 10th and 90th centiles were determined for absolute and percentage lymphocyte subset concentrations in each age group. All immunologic values were log-transformed, and regression analyses were used to assess a linear association with age. The effect of sex was explored by including sex as a term in the regression analysis and by comparing geometric means for each parameter by sex in each age group. Sex was also included as an interaction term in the regression analyses to identify any age-related effects of sex. All statistical analyses were undertaken by using Stata 10 (Stata Corp, College Station, Tex).

### Ethical approval

The study was approved by the College of Medicine Research and Ethics Committee of the University of Malawi.

## Results

### Characteristics of study subjects

We recruited 657 subjects (52-75 per group), of whom 341 (53%) were female. The median age was 53.5 months (range, 2 weeks to 92 years). Sixteen withdrew when the blood sample was not obtained at the first attempt, and 1 was of unknown age. Samples were excluded for 42 subjects infected with HIV, 17 with malaria parasitaemia, 17 taking antibiotics and 5 severely malnourished subjects. All subjects were afebrile. Twenty results failed internal quality assurance. Of the 539 subjects whose lymphocyte subset data were included in the analysis, 256 (47.5%) were male and 283 female with a sex imbalance in some groups.

### Absolute lymphocyte subset concentrations

Total leukocyte and total lymphocyte concentrations peaked at 12 to 18 months and then gradually fell by a factor of 2 and 3, respectively, to adult levels (Table I). This trend was followed by B and T lymphocytes, and CD4^+^, CD8^+^, and γδT-cell subsets, with a 10-fold difference in B-cell and 2-fold to 3-fold difference in T-cell and T-cell subset median concentrations at 12 to 18 months compared with adults. NK-cell levels varied minimally throughout life. Total leukocyte, lymphocyte, and lymphocyte subset concentrations did not fall away in old age.

### Relative lymphocyte subset values

Considering lymphocyte subset values as percentages of the total lymphocyte count removed much of the age-related variation observed with absolute concentrations (Table II), and for this reason, CD4^+^ T-lymphocyte percentages are considered a more appropriate measurement of CD4 count than absolute concentrations in children under 5 years.^10^ Nevertheless, median B-lymphocyte percentages decreased in the first 5 years of life from 30% to 22%, whereas T-lymphocyte percentages increased from 58% to 68%. CD4^+^ T-lymphocyte percentages, although ranging from 35% to 39% in children under 5 years, did not vary consistently with age. By contrast, CD8^+^ T-lymphocyte percentages increased from 19% to 25%.

As a consequence of these low CD4^+^ T-lymphocyte percentages in children under 5 years, 35% (38) of 109 children age less than 1 year had values ≤35% for this age group, the WHO threshold for immunodeficiency in HIV-infected children, with 18 meeting the criterion for mild (CD4^+^ 30% to 35%), 17 for advanced (CD4^+^ 25% to 29%), and 3 for severe (CD4^+^ < 25%) immunodeficiency.^2^ Eighteen percent (25) of 137 children age 1 to 3 years had CD4^+^ T-lymphocyte percentages ≤30%, the threshold for immunodeficiency in this age group, with 16 meeting the criterion for mild (CD4^+^ 25% to 30%), 6 for advanced (CD4^+^ 20% to 24%), and 3 for severe (CD4^+^ < 20%) immunodeficiency. Only 1 of 45 children age 3 to 5 years had CD4^+^ T-lymphocyte percentages ≤25% for this age group, the WHO threshold for immunodeficiency.

Median CD4^+^ to CD8^+^ T-lymphocyte ratio (CD4:CD8 ratio) varied between 1.4 and 2.0 across age groups. Eleven percent (60/543) of all subjects and 13% (20) of 155 children age less than 18 months had a ratio <1.0, which has been associated with HIV infection.^10^ There was little variation between T, B, and NK–lymphocyte subsets in adults (20-60 years) and the elderly (over 60 years), although the CD19^+^CD27^+^ memory B-cell population increased throughout life to a peak of 41% in old age.

### Variation of lymphocyte subsets with age and sex

Log transformation resulted in approximately normal distributions for all values except percentage memory B lymphocytes. There was very strong evidence for linear trend with age for all parameters measured (*P* < .001), except NK-lymphocyte absolute counts and CD4:CD8 ratio. There was some evidence for a quadratic relationship between age and CD4:CD8 ratio (*P* = .05). Total leukocytes and total lymphocyte concentrations, together with all absolute lymphocyte subset concentrations and B and γδT–lymphocyte percentages and CD4:CD8 ratio, decreased with age, whereas T-lymphocyte and CD4^+^ and CD8^+^ T-lymphocyte subsets and NK-lymphocyte and memory B-lymphocyte percentages increased with age (test for linear trend *P* <.001 for all parameters). There was some evidence of a quadratic rather than linear fit for absolute lymphocyte subset concentrations, with counts starting to increase in the oldest group. Using actual age, age by group, or the midpoint of age by group gave similar results.

Overall, females had significantly higher CD4^+^ T-lymphocyte percentages than males (geometric mean, 39.8% and 36.9%, respectively; *P* < .001), whereas males had higher CD8^+^ T-lymphocyte percentages than females (geometric mean, 24.15% and 22.7%, respectively; *P* = .009). As a consequence, females had higher CD4:CD8 ratios than males (geometric mean, 1.75 compared with 1.53; *P* < .001), although there was no significant difference in percentage or absolute T or B–lymphocyte concentrations or total lymphocyte count. The difference in geometric mean absolute CD4^+^ (1491 cells/μL for females compared with 1321 cells/μL for males; *P* < .001) but not CD8^+^ T-lymphocyte concentrations between the sexes was statistically significant. No age-related differences in sex were identified, and individual group sizes were too small to demonstrate statistically significant differences within each age group.

## Discussion

In this study we have enumerated lymphocyte subsets in venous blood from more than 500 healthy HIV-uninfected Malawians stratified by age. Our results show many similarities to those from studies conducted in developed countries.^11-14^ Total lymphocyte concentrations, together with total B-cell and T-cell/T-cell subset concentrations peaked in the first 2 years of life, then decreased through childhood to adulthood, whereas NK-cell concentrations were relatively constant. Lymphocyte subset values for adults were remarkably similar to those from adults in developed countries.

Total lymphocyte concentrations are higher in early childhood in Malawian children compared with children in developed countries and decrease to similar levels around 5 years of age. This difference appears to be the result of higher B-cell concentrations in early childhood in the Malawian population. Total T-cell concentrations are very similar in African and western children, but the peak median CD4^+^ T-cell concentration is lower (2.2 × 10^3^/μL compared with 2.85 × 10^3^/μL) and CD8^+^ T-cell concentration higher (1.4 × 10^3^/μL compared with 1.05 × 10^3^/μL) among Malawian children compared with children from developed countries.^11^

There are also differences in the relative sizes of lymphocyte subsets. Whereas in developed countries, T-lymphocyte percentages and B-lymphocyte percentages are stable in the first 5 years of life at around 65% and 24%, respectively, in Malawi, T-lymphocyte percentages rise from 58% to 68%, and B-lymphocyte percentages fall from 30% to 22% during this period. These findings, together with a rise in CD8^+^ T-lymphocyte percentages from 0 to 5 years, mean that CD4^+^ T-lymphocyte percentages in African children are low in the first year of life and remain relatively stable between 35% and 39% up to 5 years of age. In contrast, CD4^+^ T-lymphocyte percentages in developed countries fall from around 46% to 38% in the first 5 years. We speculate that high B-cell and CD8^+^ T-cell numbers in African children under 2 years may be associated with widespread infection with malaria and cytomegalovirus.

Although there are a lack of published reports from Africa describing variations in T, B, and NK–lymphocyte subset data with age, CD4^+^ and CD8^+^ T-lymphocyte subsets have previously been studied in African children in isolation from B and NK cells. In Nairobi, Kenya, CD4^+^ T-lymphocyte percentages in children under 1 year old were very similar to those from Malawi (39.9% up to 3 months and 36.5% from 4 to 12 months), but approximately 4% lower in children age 1 to 5 years, with median values varying from 32.9% to 35.0%.^21^ Median CD4^+^ T-lymphocyte percentages in Ugandan children under 1 year old were found to be particularly low at 31.9%, with values in children from 1 to 5 years very similar to those from Kenya (33.5%).^16^ These studies indicate that the low CD4^+^ T-lymphocyte percentages in Malawian children are not unusual for Africa.

CD8^+^ T-lymphocyte percentages were higher in Kenyan children compared with Malawian children (approximately 25% in children under 1 year old and 30% in children age 1 to 5 years),^21^ although surprisingly lower in Ugandan children (31.9% in children under 1 year old and 33.5% in children age 1 to 5 years).^16^ This finding suggests a relative expansion of B and NK–cell compared with T-cell populations in Ugandan children that cannot be confirmed because of a lack of data on these lymphocyte subpopulations. Absolute CD4^+^ and CD8^+^ T-lymphocyte subsets were broadly similar in Malawian and Kenyan^21^ children under 5 years, but lower in Ugandan children.^16^

The lack of decline in lymphocyte subset numbers among elderly Malawians suggests that this aspect of the immune system is protected from the effects of immune senescence. By contrast, a study among Italians age 4 to 106 years found decreased B and T–lymphocyte concentrations in old age,^26^ although 19% (26) of the participants were centenarians, whereas the oldest Malawian in our study was 92 years old. Our finding of increasing memory compartment of B cells with age is consistent with a previous report from Europe.^27^

As has previously been found in a developed-world population,^11^ age was highly significant in lymphocyte subset count regressions, and in this study, significance was detected with all subsets investigated both for absolute and percentage concentrations except NK-cell absolute counts and CD4:CD8 ratios. The unexpected finding from the statistical analysis was the highly significant difference in CD4^+^ and CD8^+^ T-lymphocyte percentages between the sexes, with high relative CD4^+^ counts in females and high CD8^+^ counts in males.

Reporting of sex differences in lymphocyte subset concentrations in previous studies across ages has been variable. Where given, differences have been inconsistent between studies. One western immunophenotyping study in children found a significant difference with sex only for CD4^+^ T-cell percentage,^11^ whereas a study from Ethiopia found such a difference in adults but not children.^16^ A recent study in Kenyan adults found that T, B, and NK–lymphocyte and T-lymphocyte subset absolute and percentage numbers were higher in women than men except for NK and CD8^+^ T–cell percentages.^19^ In contrast, a separate study among East and Southern African adults from a variety of sites found no significant sex differences for CD4^+^ lymphocyte counts.^20^ Our study was not designed to explore sex differences in lymphocyte subsets, and this issue warrants further investigation.

Our findings have important implications for current guidelines regarding the immunologic assessment of HIV-infected children in resource-limited settings.^2^ Although median and centile values determined in the current study were from healthy HIV-uninfected African children, the relative low CD4^+^ T-lymphocyte percentages in children under 1 year old meant that 35% of these children met the CD4 criterion for immunodeficiency in HIV-infected children. Interestingly, 10% of healthy American children under 1 year of age also met this criterion.^11^ Our finding that CD4^+^ T-lymphocyte percentages were relatively stable through 5 years of age in Malawian children contrasts with the WHO threshold values for immunodeficiency, which fall with age in a stepwise manner from 0 to 5 years. The data presented suggest that caution should be exercised when applying current guidelines for the immunologic assessment of HIV-infected African children in isolation and underline the importance of using CD4 measurements in conjunction with clinical assessment.^2^

HIV-infected children in South Africa under 12 months of age have recently been shown to have a significantly improved clinical outcome if started early on ART regardless of CD4^+^ T-lymphocyte percentage.^28^ This has led to a change in national policy and guidelines in Malawi^29^ as well as the US National Institutes of Health^30^ and European^31^ guidelines on the management of HIV-infected children such that all children under 12 months of age with proven infection should be commenced on ART. Therefore, the findings from the current study are unlikely to affect treatment of children in this age group.

A CD4:CD8 T-lymphocyte ratio less than 1.0 is associated with HIV infection and has been proposed as a parameter for the diagnosis of HIV in African children under 18 months old,^32^ where results of HIV rapid tests can be complicated by maternal antibody to HIV.^10,32^ With decreased CD4^+^ and increased CD8^+^ T-lymphocyte concentrations in young children in this study compared with results from developed countries, 13% of Malawian children under 18 months had a CD4:CD8 ratio <1.0. Therefore, a CD4:CD8 ratio <1.0 is not specific for HIV infection, and this parameter should be used with caution in HIV diagnosis.

In conclusion, our findings provide a comprehensive description of principle lymphocyte subsets and T-lymphocyte subsets with age in a healthy African population. The findings suggest that caution should be exercised when assessing the immunologic status of HIV-infected African children using current WHO guidelines and serve as a reminder to clinicians to take into consideration the clinical status of the child alongside laboratory parameters. Further studies from elsewhere in Africa are required to confirm whether the low CD4^+^ T-lymphocyte percentages found in HIV-uninfected children in this study in Malawi and in previous studies from Kenya and Uganda are a consistent finding throughout the continent. In addition, the sex variation in CD4^+^ T-lymphocyte percentage and absolute concentrations deserves further investigation, in particular to determine whether higher values in females occur at all ages.

**Clinical implications**

CD4^+^T-lymphocyte percentages in young African children are low compared with those in children from developed countries, and this must be taken into account when assessing the severity of HIV-related immunodeficiency in these children.

## Figures and Tables

**Table I:** Absolute lymphocyte subset concentrations by age in venous blood from healthy Malawians

Age groups												
Subset	0-6 mo (n = 58)	6-12 mo (n = 51)	12-18 mo (n = 46)	18-24 mo (n = 45)	2-3 y (n = 46)	3-5 y (n = 42)	5-10 y (n = 52)	10-15 y (n = 49)	15-20 y (n = 51)	20-60 y (n = 49)	>60 y (n = 50)	*P* value for linear trend∗
Male:female	21:37	20:31	27:19	29:16	27:19	21:21	26:26	14:35	26:25	23:26	22:28	
Total leukocytes	9.4	9.7	10.95	9.4	8.0	8.2	6.5	5.7	5.2	5.0	5.65	<.001
	(6.2, 15.3)	(8.1, 14.2)	(7.1, 15.7)	(6.5, 13.2)	(6.5, 11.5)	(6.6, 9.8)	(4.7, 10.8)	(3.9, 8.1)	(3.7, 7.3)	(3.9, 7.2)	(4.35, 6.95)	
Total lymphocytes	5.45	6.3	7.0	5.4	4.25	3.7	3.1	2.8	2.1	2.0	2.4	<.001
	(3.7, 9.2)	(4.5, 9.6)	(4.4, 9.3)	(3.55, 7.4)	(2.7, 6.0)	(2.6, 5.1)	(2.2, 4.9)	(1.9, 3.8)	(1.7, 3.2)	(1.4, 2.9)	(1.75, 2.95)	
B lymphocytes	1.5	1.8	1.9	1.3	1.0	0.7	0.5	0.4	0.3	0.2	0.2	<.001
(CD19^+^)	(0.7, 2.8)	(1.2, 2.9)	(1.0, 3.5)	(0.7, 2.4)	(0.5, 1.5)	(0.4, 1.3)	(0.3, 0.8)	(0.2, 0.7)	(0.2, 0.5)	(0.1, 0.3)	(0.1, 0.4)	
T lymphocytes	3.4	3.9	4.0	3.3	2.9	2.4	2.1	1.9	1.5	1.4	1.6	<.001
(CD3^+^)	(2.2, 6.1)	(2.7, 5.6)	(2.1, 5.5)	(2.4, 4.8)	(1.7, 4.5)	(1.8, 3.7)	(1.4, 3.4)	(1.3, 2.7)	(1.1, 2.4)	(0.9, 2.1)	(1.2, 2.2)	
CD4^+^ T lymphocytes	2.0	2.2	2.2	1.8	1.7	1.4	1.2	1.1	0.9	0.8	1.0	<.001
(CD3^+^CD4^+^)	(1.3, 4.2)	(1.6, 3.3)	(1.2, 3.9)	(1.2, 2.7)	(1.0, 2.6)	(0.9, 2.0)	(0.8, 2.1)	(0.8, 1.7)	(0.6, 1.2)	(0.5, 1.2)	(0.6, 1.3)	
CD8^+^ T lymphocytes	1.0	1.4	1.4	1.3	1.0	0.9	0.8	0.7	0.6	0.6	0.5	<.001
(CD3^+^CD8^+^)	(0.6, 2.1)	(0.8, 2.3)	(0.8, 2.3)	(0.7, 2.0)	(0.6, 1.6)	(0.6, 1.5)	(0.5, 1.2)	(0.4, 1.1)	(0.4, 1.0)	(0.3, 1.0)	(0.3, 0.9)	
γδT lymphocytes	0.2	0.2	0.3	0.2	0.2	0.2	0.2	0.1	0.1	0.1	0.1	<.001
(CD3^+^TCRγδ^+^)	(0.1, 0.4)	(0.1, 0.5)	(0.1, 0.7)	(0.1, 0.4)	(0.1, 0.4)	(0.1, 0.4)	(0.1, 0.3)	(0.1, 0.3)	(0.1, 0.2)	(0.0, 0.2)	(0.0, 0.1)	
NK cells	0.4	0.4	0.4	0.3	0.3	0.3	0.3	0.3	0.3	0.3	0.4	.54
(CD3-CD16/56^+^)	(0.2, 1.0)	(0.2, 0.7)	(0.2, 1.2)	(0.2, 0.7)	(0.2, 0.8)	(0.1, 0.6)	(0.1, 0.9)	(0.1, 0.6)	(0.1, 0.5)	(0.1, 0.7)	(0.2, 0.8)	

Values are medians (10th and 90th centiles) × 10^3^ cells/μL venous blood.

∗ Calculated by using actual ages in months.

**Table II:** Percentage lymphocyte subset concentrations by age in venous blood from healthy Malawians

Age groups												
Subset	0-6 mo (n = 58)	6-12 mo (n = 51)	12-18 mo (n = 46)	18-24 mo (n = 45)	2-3 y (n = 46)	3-5 y (n = 42)	5-10 y (n = 52)	10-15 y (n = 49)	15-20 y (n = 51)	20-60 y (n = 49)	>60 y (n = 50)	*P* value for linear trend‡
Male:female	21:37	20:31	27:19	29:16	27:19	21:21	26:26	14:35	26:25	23:26	22:28	
B lymphocytes	29	30	27	25	22	22	17	14	13	9	9	<.001
(CD19^+^)	(16, 38)	(21, 38)	(18, 39)	(17, 36)	(13, 27)	(15, 29)	(12, 24)	(11, 21)	(8, 18)	(6, 14)	(5, 16)	
Memory B cells	4	8	10	17	22	22	28	28	31	38	41	<.001
(CD19^+^CD27^+^)^∗^	(3, 7)	(4, 13)	(6, 19)	(10, 25)	(11, 30)	(14, 31)	(15, 40)	(16, 40)	(18, 44)	(19, 59)	(24, 64)	
T lymphocyte	58	61	61	66	66	68	71	71	71	73	69	<.001
(CD3^+^)	(50, 72)	(52, 71)	(48, 70.5)	(54, 74)	(61, 76)	(58, 77)	(59, 79)	(59, 79)	(61, 77)	(63, 83)	(57, 78)	
CD4^+^ T lymphocytes	39	36	35	38	39	37	40	41	41	41	42	<.001
(CD3^+^CD4^+^)	(29, 49)	(27, 44)	(23, 45)	(27, 49)	(32, 46)	(31, 47)	(29, 49)	(35, 52)	(31, 48)	(29, 51)	(33, 52)	
CD8^+^ T lymphocytes	19	22.5	22	24	24	25	25	24	25	28	23	.002
(CD3^+^CD8^+^)	(13, 25)	(15, 32)	(16, 35)	(15, 32)	(18, 32)	(18, 34)	(18, 32)	(17, 34)	(19, 35)	(20, 37)	(17, 35)	
γδT lymphocytes	3	4	4	4	4	5	5	6	5	5	3	.001
(CD3^+^TCRγδ^+^)	(1, 5)	(2, 8)	(2, 7)	(3, 7)	(3, 8)	(3, 9)	(3, 9)	(3, 9)	(3, 9)	(2, 10)	(1, 8)	
NK cells	9	6	6	7	9	8	8	9.5	12	16	17	.001
(CD3-CD16/56^+^)	(4, 16)	(4, 11)	(3, 15)	(3, 12)	(5, 15)	(4, 15)	(3, 22)	(5, 19)	(5, 23)	(6, 26)	(8, 32)	
CD4:CD8	2.0	1.6	1.6	1.5	1.6	1.5	1.6	1.8	1.6	1.4	1.8	.98
T-lymphocyte ratio^†^	(1.3, 3.6)	(0.9, 2.7)	(0.8, 2.9)	(1.0, 2.6)	(1.0, 2.3)	(1.0, 2.4)	(1.1, 2.3)	(1.1, 2.6)	(1.0, 2.3)	(0.9, 2.3)	(1.1, 2.8)	

Values are medians (10th and 90th centiles) presented as percentage of total lymphocyte concentration, except ^∗^as percentage of total B-cell concentration, and ^†^as ratio of CD4^+^ T lymphocytes to CD8^+^ T lymphocytes.

‡ Calculated by using actual ages in months.
